# Vertical transmission of porcine circovirus-like virus P1 in BALB/c mice

**DOI:** 10.1186/s12917-023-03669-2

**Published:** 2023-07-28

**Authors:** Shaoyang Sheng, Lin Guan, Jianping Xie, Qi Xiao, Libin Wen, Kongwang He

**Affiliations:** 1 Institute of Veterinary Medicine, Jiangsu Academy of Agricultural Sciences, Nanjing, 210014 P. R. China; 2 Key Laboratory of Veterinary Biological Engineering and Technology, Ministry of Agriculture, Nanjing, 210014 P. R. China; 3 Jiangsu Co-innovation Center for Prevention and Control of Important Animal Infections Diseases and Zoonoses, Yangzhou University, Yangzhou, 225009 P. R. China; 4 Jiangsu Key Laboratory for Food Quality and Safety - State Key Laboratory Cultivation Base of Ministry of Science and Technology, Nanjing, 210014 P. R. China; 5 GuoTai (Taizhou) Center of Technology Innovation for Veterinary Biologicals, Taizhou, 225300 P. R. China

**Keywords:** Porcine circovirus-like virus P1, Vertical transmission, BALB/c mice

## Abstract

**Background:**

Porcine circovirus-like virus P1 is the animal virus with the smallest genome discovered so far, and it has become widely distributed in the Chinese mainland in recent years.

**Results:**

In this study, a BALB/c mouse model was used to reveal P1 infection in female reproductive systems and the vertical transmission of the virus. The female reproductive system, including the ovary and uterus, was harvested on day 14 postinfection and examined for pathological lesions. One-day-old mice without colostrum born from infected or uninfected mothers were collected, and P1 virus distribution in the different organs was investigated. During the trials, all the mice showed no clinical symptoms or gross lesions. However, stillbirth did occur in groups infected with the P1 virus. P1 nucleic acid was detected in the heart, liver, spleen, lung, kidney, and brain tissues of 1-day-old mice born from infected mice. Microscopic lesions in P1-infected female mice were characterized by necrosis of the ovarian follicular granulosa cells and abscission, follicular atresia, necrosis of the endometrial epithelial and uterine glandular epithelial cells, and hyperplasia of the squamous endometrial epithelium. The spermatocytes in the seminiferous tubules of the infected male mice were disorderly arranged, and the germ and Sertoli cells were shed, necrotic, and decreased in number. Immunohistochemical results identified P1-positive particles in the nucleus and cytoplasm of cells from the ovary and uterus of female mice.

**Conclusions:**

This study shows that the P1 virus could cause pathological damage to the reproductive system of female mice and could be transmitted vertically.

## Background

Porcine circovirus-like virus P1, which by far has the smallest known circular DNA genome, is a small, nonenveloped virus that is widespread in pig populations in many provinces in mainland China. The genome length of the P1 virus is only 648 or 649 nucleotides and is highly homologous with that of the porcine circovirus type 2 (PCV 2) but is only one-third of the length of the PCV2 [1, 2]. PCV2, a member of the *Circovirus* genus, is the smallest, single-stranded, circular DNA virus that can replicate autonomously in mammalian cells, and is considered a primary causative agent of porcine circovirus-associated diseases (PCVADs), such as porcine post-weaning multisystemic wasting syndrome (PMWS) [3]. Current research shows that the P1 virus can naturally infect pigs, cattle, goats, rabbits, dogs, cats, and yaks, and artificially infect laboratory mice [1, 4–7]. Because it was associated with PMWS, which is characterized by progressive weight loss and jaundice, the P1 virus has been linked with other emerging diseases in pigs, such as abortion and congenital tremor [8–10], indicating that the P1 virus may have a wider pathogenic spectrum. Evidence associating PCV1, identified in the porcine kidney cell line PK-15 in the 1970s, PCV2, and PCV3 with vertical transmission has also been reported [11–13]. The detection of the P1 virus rather than the porcine parvovirus (PPV), porcine reproductive and respiratory syndrome virus (PRRSV), classical swine fever virus (CSFV), Japanese encephalitis virus (JEV), pseudorabies virus (PRV) and PCV2 in samples of aborted porcine fetuses in China also indicates vertical transmission of the P1 virus [9]. However, to date, there has been no direct experimental evidence that the P1 virus can infect the fetus.

Herein, we examined whether P1 virus infection can cause pathological damage to the female reproductive system and be vertically transmitted to newborns. Hence, we performed two key experiments. In experiment 1, we assessed the effect of the P1 virus on the female reproductive system, and in experiment 2, we determined the vertical transmission of the P1 virus. Considering the high infection rate of circoviruses in conventional porcine, the BALB/c mice were used as a model in this study.

## Results

**Fig. 1:**
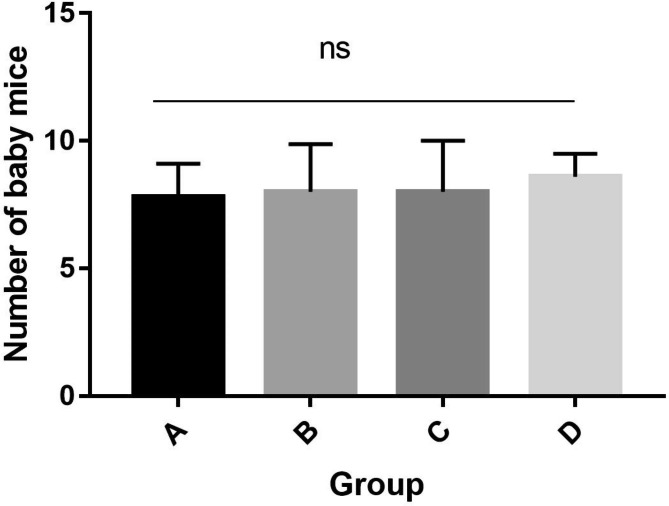
The effect of porcine circovirus-like virus P1 on the number of mice born per litter. **(A)** All mice were inoculated with a cloned genomic DNA fragment of the P1 virus **(B).** Only female mice were inoculated with a cloned genomic DNA fragment of the P1 virus **(C).** Only male mice were inoculated with a cloned genomic DNA fragment of the P1 virus **(D).** All mice were inoculated with the pSK vector. The data represent the means ± SDs. ns, no significance

All mice were clinically healthy and had no clinical signs for the entire duration of the experiment. Female mice in group A gave birth to 9, 9, 8, 6, and 7 mice per litter. Female mice in group B gave birth to 7, 6, 10, 10, and 7 mice per litter. Group C female mice gave birth to 9, 9, 5, 10, and 7 mice per litter. Female mice in group D gave birth to 8, 8, 8, 10, and 9 mice per litter. Although there was no significant difference in the average number of offspring produced by female mice in the four groups, one female mouse in group A and two female mice in group B each gave birth to one stillborn mouse (Fig. 1).

All 12 selected offspring mice in group D tested negative using PCR and qPCR detection of P1 DNA in their tissues postmortem. Each litter of the offspring mice in groups A, B, and C was P1 DNA-positive as determined by the presence of the P1 DNA in various tissues using PCR. Among the 13 randomly selected mice in Group A, eight were positive for the P1 virus; of these, the hearts, livers, and lungs of eight mice, the spleens of three mice, and the kidneys and brains of five mice were positive for the P1 virus. Out of 12 randomly selected mice in Group B, eight were positive for the P1 virus; in this group, the hearts, livers, and lungs of eight mice, the spleens of six mice, the kidneys of four mice, and the brains of two mice were positive for the P1 virus. In Group C, 6 of 12 randomly selected mice were positive for the P1 virus, and the hearts, livers, kidneys, and lungs of six mice, the spleens of three mice, and the brains of two mice were positive for the P1 virus. The results show that the heart, liver, and lungs were the main organs with the highest virus detection rate (Table 1).

**Table 1:** Detection and distribution of P1 viral DNA using PCR in the control and P1-inoculated mice via the intramuscular route

Group	P1-inoculated mice	Number of pups (including stillborn)	No. positive/no. tested for:						
			Heart	Liver	Spleen	Lung	Kidney	Brain	
A B C D	All Female Male None	39 40 40 43	8/13 8/12 6/12 0/12	8/13 8/12 6/12 0/12	3/13 6/12 3/12 0/12	8/13 8/12 6/12 0/12	5/13 4/12 6/12 0/12	5/13 2/12 2/12 0/12	

**Fig. 2:**
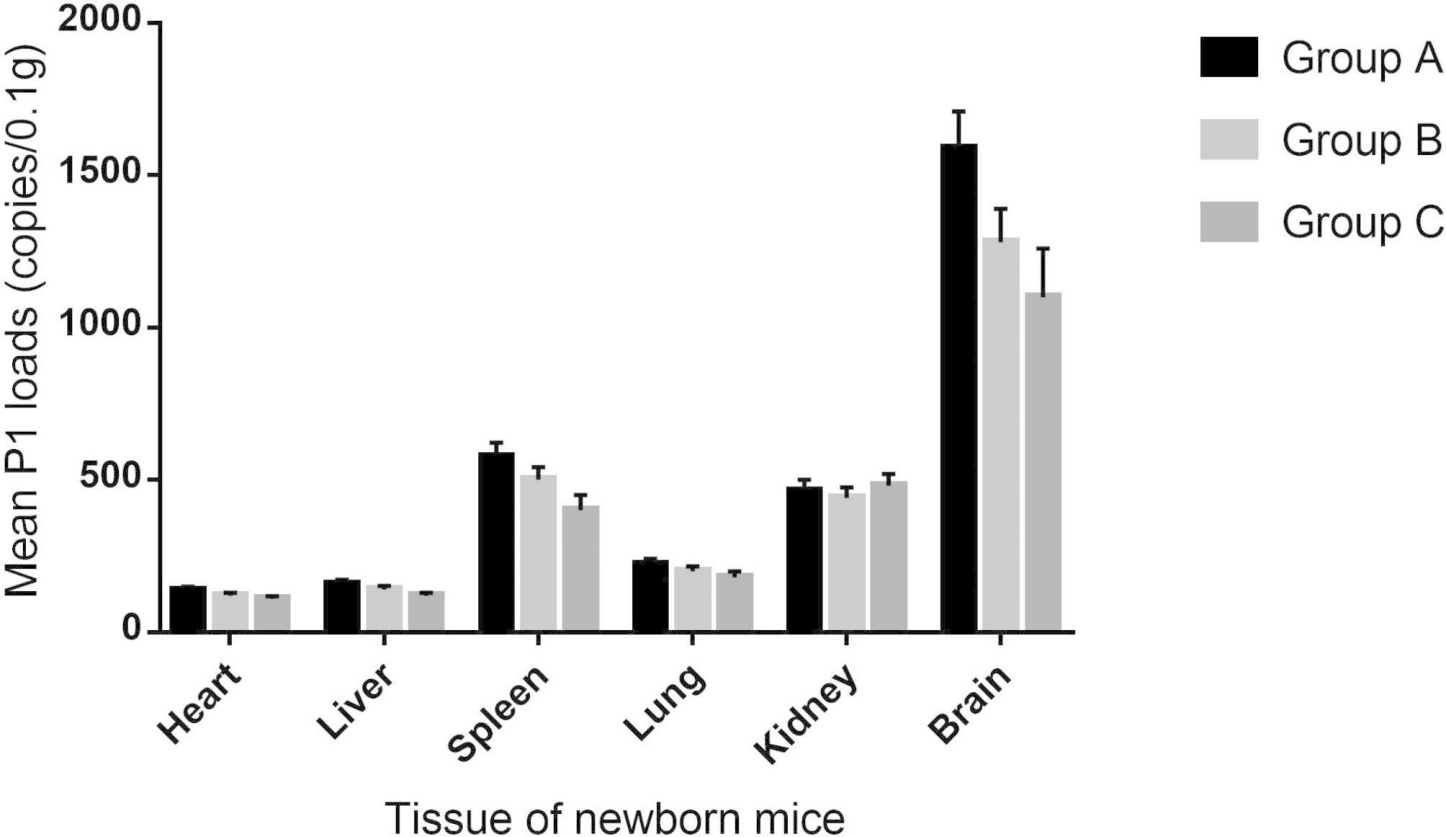
Viral loads detected by qPCR in different tissues from the neonatal mice of groups A to C. Values are represented as the mean ± SD of P1 genome copy numbers/0.1 g of tissue

In group A, all the heart tissues positive for the P1 genomic DNA had a mean of 139 genomic copies/0.1 g of tissue. The P1 DNA was detected in the liver and lungs at a level of 159 and 223 copies/0.1 g of tissue, respectively. The mean number of P1 DNA copies in the spleen and kidney was 577 and 463 copies/0.1 g, respectively. The P1 DNA was found in brain tissue with a higher genomic load than in the other tissues (1590 copies/0.1 g of tissue). There was no significant difference in the distribution profile of the P1 virus and viral load in the same type of tissues between the two inoculated groups B and C, and the mean number level of P1 DNA copies in the P1-positive tissues remained at similar genomic loads compared with those of group A (Fig. 2).

**Fig. 3:**
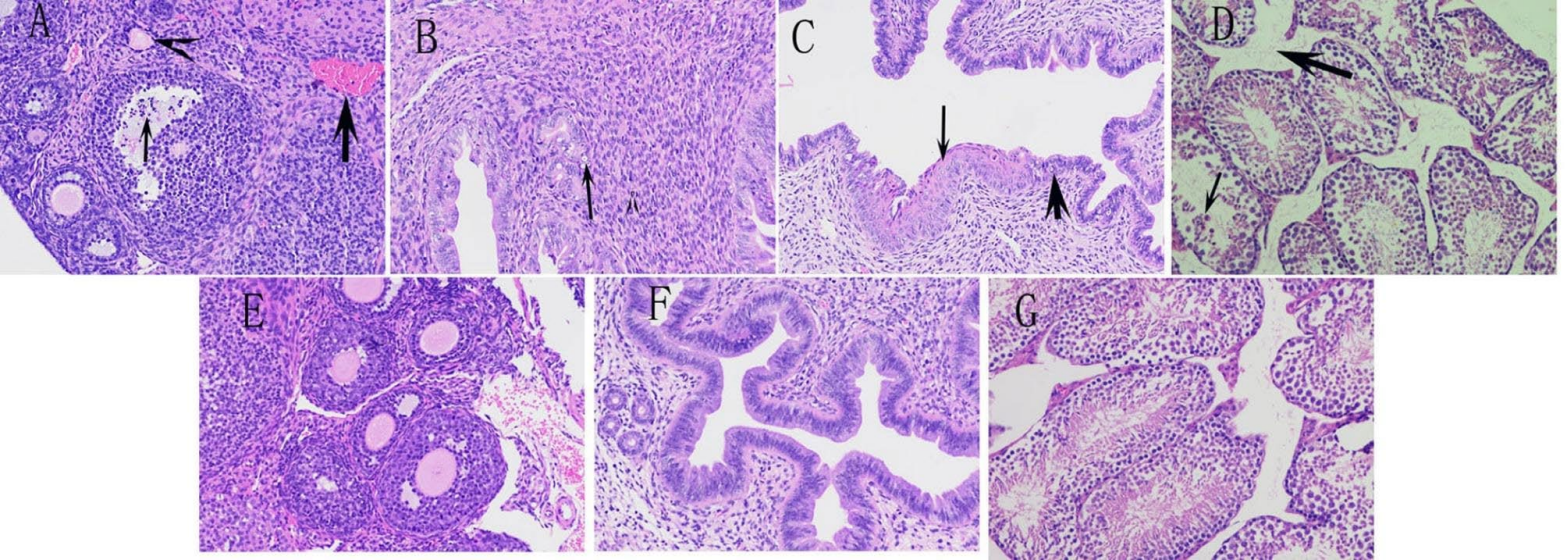
Histopathology of tissues from the intramuscularly challenged mice, stained with hematoxylin and eosin; 200x magnification. **(A).** Ovary with necrotic follicular granulosa cells and abscission (thin arrow), follicular atresia (arrowhead), and congestion of interstitial veins (thick arrow). **(B).** Necrosis of glandular epithelial cell (arrow). **(C).** Necrosis of the endometrial epithelial cells (thick arrow) and endometrial squamous hyperplasia (thin arrow). **(D).** The spermatozoa in the seminiferous tubules of the infected male mice were disorderly arranged (thin arrow) and had interstitial edema (thick arrow). **(E)** Normal ovary from a control mouse. **(F)** Normal uterus from the control group. **(G)** Normal testis from the control group

No gross lesions were observed in the female mice in experiment 1 and the male mice in experiment 2, either with inoculation of P1 viral molecular clones or of the empty vector. The histopathological observation showed that the ovaries and the uterus of the female mice in group P had pathological changes to varying degrees. In the ovary, several follicular granulosa cells were necrotic and detached; follicular atresia, abnormal follicular morphology, irregular oocyte morphology, and congestion of interstitial veins were observed. In the uterus, the necrosis of endometrial epithelial cells increased significantly, endometrial squamous metaplasia was seen, and necrosis of the epithelial cells of the uterine gland was seen. There was no obvious pathological change in the ovary and uterus of the group N mice. Microscopic lesions in the testes of mice of group D in experiment 2 were unremarkable. The seminiferous tubules of the testes were normal, round or oval, regularly distributed, and the structure of the seminiferous epithelium was complete, including the spermatogenic and Sertoli cells at all stages. There were abundant spermatozoa in some seminiferous tubules. Compared with the normal testis, the spermatozoa in the seminiferous tubules of the P1-infected male mice were disorderly arranged, and the primary spermatocytes, secondary spermatocytes, and Sertoli cells were shed, necrotic, and decreased in number with interstitial edema (Fig. 3).

**Fig. 4:**
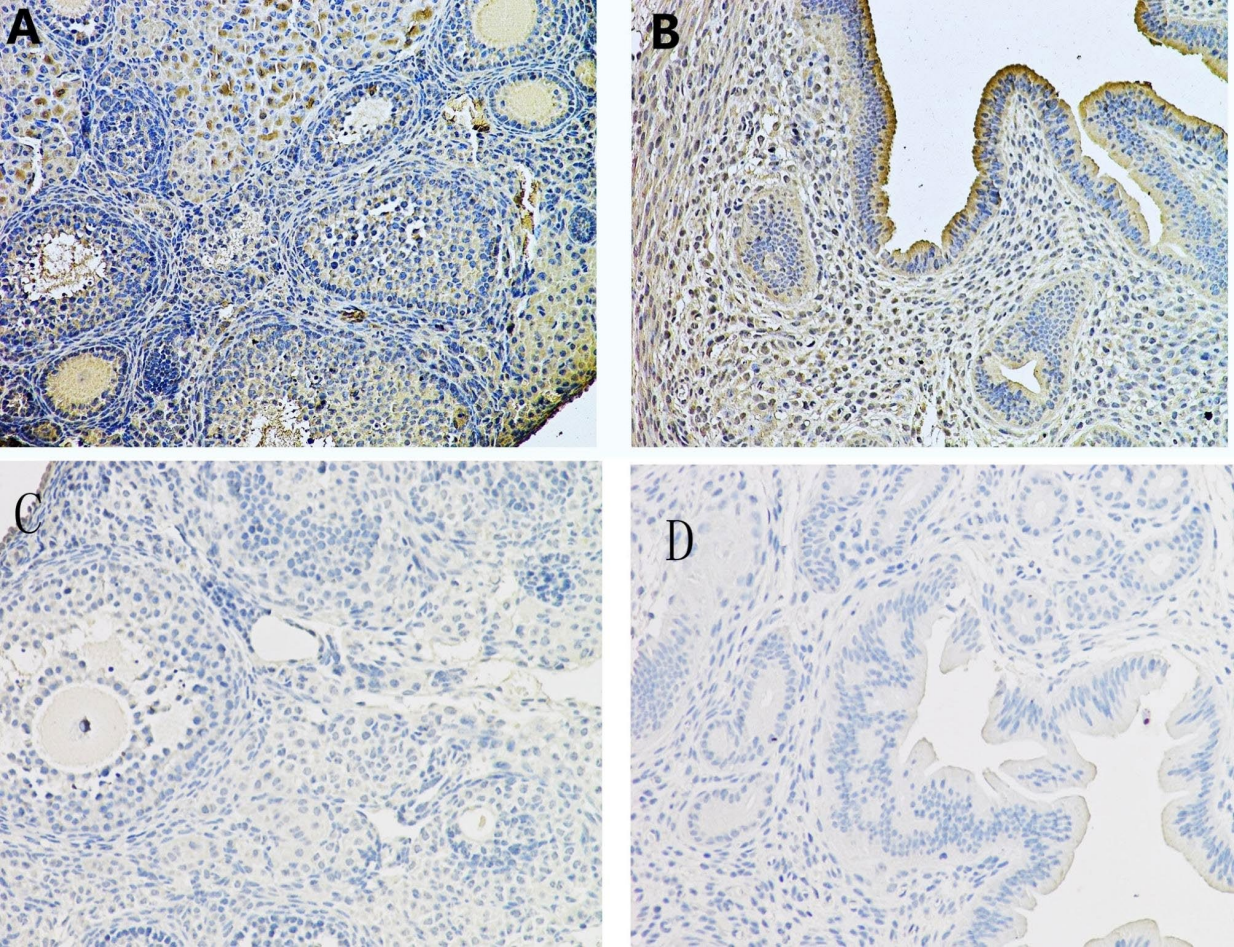
Immunohistochemical staining of mice ovary and uterus tissues with a polyclonal antibody. P1 antigen-positive cells are stained brown. Many cells positive for the P1 antigen were observed in the ovary **(A)** and uterus **(B)** of the P1-inoculated mice. **(C)** Normal ovary from a control mouse. **(D)** Normal uterus from the control group

P1 antigen was detected in the nucleus and cytoplasm of cells from the ovary and uterus of four/five female mice using immunohistochemistry. Most of the antigen particles were in the cytoplasm, rather than in the nucleus. In the ovary, P1 viral antigen was mainly present in the surface epithelium and interstitial cells; strong staining of the epithelial cells was seen in the uterus (Fig. 4).

## Discussion

The P1 virus was discovered nearly 20 years ago, but there is still no suitable cell line for its propagation [14]. Infectious molecular clones of the virus or rescue virus can be used in the research of virus pathogenesis. Similar to PCV2, the double-copy tandem molecular clone of the P1 genome was infectious in vitro and in vivo [2, 8, 15]. Previous studies have reported that the infectious clone P1 can infect mice through intramuscular injection [16]. Compared with viruses, the advantages of using infectious cloning of viruses are that there is no need to culture a virus in a cell, determine the virus titer, and worry about external virus contamination. Although some DNA plasmids are distributed across a wide range of tissues in injected individuals for up to several weeks, it is unclear whether the detected copy numbers of the viral genome are from the plasmid or the rescued virus [17, 18]. However, this problem can be solved by detecting the virus antigen, determining whether there are antibodies specific to the virus, and determining whether the number of copies of the virus genome increases. This study used the infectious clone of the P1 virus to study the damage caused by the virus to the reproductive system of female mice and whether the virus could be vertically transmitted to the next generation of mice through intramuscular injection.

Our study shows that the P1 virus can replicate and cause lesions in the reproductive system of female mice, causing changes in the ovaries and uterus. These results are correlated with the detection of the P1 virus in the reproductive system using IHC. In addition, histopathological changes in the testes of mice infected with P1 virus showed severe disorganization of seminiferous tubules, and necrosis and devoid of spermatic cells along with interstitial edema.

Challenged mice in groups A and B gave birth to stillborn fetuses. The P1 virus was detected in the tissues and organs of offspring mice born from female mice inoculated with the molecular cloned P1 virus in groups A and B to varying degrees, indicating that the virus has characteristics of vertical transmission. Although the group C female mice were not inoculated with the molecular clone of the P1 virus, the male mice in the same litter were inoculated with this, and the virus was detected in the tissues and organs of their offspring. The P1 virus was detected in multiple tissues, and the heart, liver, and lungs were the main targets of P1 viral infection. It remains unknown whether female mice were infected with the P1 virus horizontally via shedding of the infectious virus by cohabiting male mice, or vertically via the semen of male mice. Previous results have shown that PCV2 may be shed intermittently in boar semen [19]. The study also demonstrated that artificial insemination of the semen of healthy pigs mixed with PCV2 and transferred into healthy sows can lead to reproductive failure in sows and sows giving birth to stillborn and mummified fetuses [20]. Whether the P1 virus, like the PCV2, can be transmitted through semen remains to be elucidated in further studies.

## Conclusion

In conclusion, we demonstrated in an animal model that the P1 virus not only causes pathological damage to the reproductive system of female mice but can be transmitted to the next generation through maternal mice. This suggests that the P1 virus can be efficiently transmitted by the vertical route and is clinically associated with sow reproductive failure.

## Materials and methods

### Animal experimental design

The healthy BALB/c mice used in this study were purchased from a specific-pathogen-free (SPF) colony (Shanghai Sippr-BK laboratory animal Co. Ltd. China). Before the animal experiments, the PCV1/2/3 or P1 antibody and/or antigen detection were tested to ensure that the mice were PCV1/2/3 or P1 negative. In experiment 1, 10 6-week-old BALB/c female mice were randomly divided into two groups, five in each group (P and N). In experiment 2, 40 6-week-old BALB/c mice were randomly assigned to four groups of 10 mice each (A, B, C, and D) with a 1:1 male:female population. All mice in groups P and A, female mice in group B, and male mice in group C were injected via the intramuscular route with a plasmid containing P1 DNA constructed as described previously, and the injection dose was 100 µL/100 µg per mouse [16]. The remaining mice were inoculated intramuscularly with the pSK vector. Each group of mice in experiment 2 was immediately divided into five cages for breeding after inoculation, and each cage contained a female and a male randomly selected and housed in an experimental barrier facility at the Jiangsu Academy of Agricultural Sciences. Two mice in each cage were allowed to mate naturally and were left together until the pups were born. The mice were observed daily for changes in appetite, water, and mental state, and the births were closely monitored. The number and vitality of the offspring were recorded.

### Sample collection

All mice in experiment 1 were euthanized on day 14 postinjection. Samples of the ovary and uterus were collected and fixed in 10% neutral buffered formalin for immunohistochemistry (IHC) and pathological analysis. After approximately 21 days of gestation, all newborn mice without colostrum were euthanized. At least two newborn mice were randomly selected from each litter, including stillborn mice, for P1 virus detection. In addition, the testicles of the male parent mice in groups C and D in experiment 2 were also collected for pathological analysis.

The mice were euthanized using an intraperitoneal injection of three times the lethal dose of pentobarbital.

Postmortem tissue samples (heart, spleen, liver, lung, kidney, and brain) were collected. The organ tissues were homogenized in 10 volumes (1:10, w/v) of 0.01 M PBS (pH 7.2) using a Polytron Tissumizer (2◊1 min on, 1 min rest). Homogenates were centrifuged at 2000◊g for 5 min at 4 °C and the supernatant was used for PCR testing for the P1 virus.

The animal study protocol was approved by the Committee on Ethics of Animal Experiments of the Institute of Veterinary Medicine, Jiangsu Academy of Agricultural Sciences (JAAS No 20,100,604), and conformed to the Jiangsu Province Animal Regulation guidelines (Government Decree No 45).

### PCR and qPCR detection of P1 nucleic acid in different organs or tissues

Viral DNA was extracted using a commercial kit (TIANDZ, China) according to the manufacturer’s protocol. A set of P1-specific primers (F: 5’-TCGTATATACTGTTTTCGAACGC-3’, R: 5’-CCAGGACTACAATATCCGTGTAA-3’) were used to amplify the full length of the P1 genome via conventional PCR. PCR was performed with an initial denaturation at 95 °C for 3 min, followed by 40 cycles of 95 °C for 30 s, 56 °C for 30 s, and 72 °C for 45 s, with a final extension at 72 °C for 5 min. The PCR products were analyzed on a 1.5% agarose gel using electrophoresis, and bands of the expected size were visualized using an ultraviolet transilluminator. All PCR amplification products were confirmed using sequencing.

P1 genomic load in the tissues (heart, spleen, liver, lung, kidney, and brain) was also evaluated using quantitative real-time PCR with two primers (the forward primer 5′- GAGAGGCGGGTGTTGAAGAT-3 ′ and the reverse primer 5′- AAGACCCCCCACTTAAACCC − 3 ′) as previously reported [21].

### Histopathology and immunochemical staining

The formalin-fixed samples (ovary, uterus, and testis) were dehydrated and embedded in paraffin wax in a single block per mouse. The paraffin-embedded tissues were sectioned at 4 μm thickness, dewaxed with xylol, hydrated with ethyl alcohol, and then stained with hematoxylin and eosin (HE) for histopathological examinations. Serial sections from the ovary and uterus were stained using IHC and a P1 rabbit polyclonal antibody as described previously [8].

### Statistical analysis

All data are presented as the means ± standard deviation (SD), which were analyzed using a nonparametric one-way analysis of variance (ANOVA) followed by the least significant difference (LSD) test for multiple group comparison; p < 0.05 was regarded as significant. Graphs were generated using GraphPad Prism 8 software (GraphPad Software, Inc., La Jolla, CA, USA).

## Data Availability

The datasets generated and/or analysed during the current study are available in the GenBank repository (EF514716).

## Abbreviations

P1 virus: Porcine circovirus-like virus P1
PCV 2: Porcine circovirus type 2
PCVADs: Porcine circovirus-associated diseases
PMWS: Porcine post-weaning multisystemic wasting syndrome
PPV: Porcine parvovirus
PRRSV: Porcine reproductive and respiratory syndrome virus
CSFV: Classical swine fever virus
JEV: Japanese encephalitis virus
PRV: Pseudorabies virus
SPF: Specific-pathogen-free
IHC: Immunohistochemistry
HE: Hematoxylin and eosin
SD: Standard deviation
ANOVA: Analysis of variance
LSD: Least significant difference
